# Editorial: Clinical High Risk for Psychosis: From Epidemiological Findings to Neurobiological Underpinnings of Treatment Response and Outcome

**DOI:** 10.3389/fpsyt.2021.790810

**Published:** 2021-11-18

**Authors:** Chantal Michel, Maximus Berger, Nilufar Mossaheb, Jochen Kindler

**Affiliations:** ^1^University Hospital of Child and Adolescent Psychiatry and Psychotherapy, University of Bern, Bern, Switzerland; ^2^University Hospital of Psychiatry and Psychotherapy, University of Bern, Bern, Switzerland; ^3^Clinical Division of Social Psychiatry, Department of Psychiatry and Psychotherapy, Medical University of Vienna, Vienna, Austria

**Keywords:** clinical high risk (CHR) for psychosis, epidemiology, precision medicine, cognitive remediation therapy, biomarkers

The majority of first-episode psychotic disorders are preceded by a prodromal phase in which a multitude of clinical high risk (CHR) symptoms, other mental health problems, and psychosocial deficits may occur, offering a window of opportunity for early detection and intervention (1, 2). This phase offers an excellent starting point for indicated prevention that, currently, aims at reducing CHR symptoms and, thereby, preventing transition to frank psychosis (3). Two major sets of CHR criteria are used for the assessment of this state: (i) ultra-high risk (UHR) criteria, i.e., attenuated (APS) or brief (limited) intermittent psychotic symptoms [B(L)IPS] and genetic risk and functional decline (GRFD); and (ii) basic symptom (BS) criteria, i.e., cognitive disturbances (COGDIS) and cognitive-perceptive basic symptoms (COPER) (1, 2). The two symptomatic UHR criteria APS and B(L)IPS include positive symptoms of psychosis where some insight into their abnormal nature is still present (4). BSs are subtle, subjectively experienced subclinical disturbances in mainly perception and cognition where insight is also present, which can usually be assessed from age eight onwards (5).

The aim of this Research Topic is to highlight and promote recent research focusing on the epidemiology and neurobiology of CHR states and psychosis onset, new treatment options as well as the consequences on patients' Quality of Life. Four original articles and one opinion piece were included in the Research Topic presenting new findings in different areas.

Joa et al. present 2-year follow-up findings of the Norwegian Prevention of Psychosis study, a large scale campaign targeting young people at UHR for psychosis that aimed to detect help-seeking individuals fulfilling UHR criteria through systematic early detection strategies. While these efforts did not lead to detecting the amount of UHR individuals predicted by the population statistics, they provide relevant lessons for future studies regarding recruitment and retention processes of the at-risk population. Furthermore, they present negative symptoms as predictors for psychosis conversion, underlining the importance of adding negative-like symptoms and social withdrawal to the focus of early detection.

Paolo Fusar-Poli's comment briefly introduces the “Dynamic ElecTronic hEalth reCord deTection (DETECT)” a precision medicine approach applying artificial intelligence to predict conversion of psychosis based on electronic health records using demographic and medical events. More importantly, he provides rationales for the need and efficacy of modern early detection approaches and takes a stand for evidence-based preventive medicine.

A promising peripheral biomarker indicating autonomic (dys-)functions and showing complex interactions with prefrontal cortical areas is resting state heart rate. Kocsis et al. demonstrate increased heart rate in individuals with CHR for psychosis that is associated with symptom severity and distress. The neurobiological roots of alterations in autonomic functioning in the CHR state have yet to be determined.

Steinmann et al.'s article focuses on characterizing sexual dimorphism of language areas in the brain by investigating the asymmetry of four white matter tracts relevant to verbal working memory in CHR patients compared to healthy controls. This study suggests increased rightward asymmetry of the superior longitudinal fascicule in CHR females. This finding of sexual dimorphism in white matter asymmetry in a language-related area of the brain in CHR highlights the need for a deeper understanding of the role of sex in the CHR states. Future work investigating early sex-specific pathophysiological mechanisms, may lead to the development of novel personalized treatment strategies aimed at preventing transition to a more chronic and difficult-to-treat disorder.

The findings of a randomized clinical trial of cognitive remediation therapy vs. treatment-as-usual were reported by Kristensen et al.. This report was an interesting investigation on white matter organization before and after 12 h of cognitive training—a therapy which previously has been shown to improve cognition and functional outcome in schizophrenia—in a large sample of UHR individuals aged 18–40 years. They found no effects of remediation therapy on white matter organization. However, studies on biological markers of treatment effects are relevant as these biomarkers may help to improve our precision for prediction and the knowledge on origins and progression of a disease—which extends far beyond treatment outcomes.

In conclusion, the results of the articles included in this Research Topic emphasize the need for various strategies and tools for the early detection of psychosis. While there is an ongoing discussion about the relevance and the specificity of the UHR criteria and of the at-risk population as a group, the findings presented in this Research Topic not only help to foster research on the characterization of CHR individuals with respect to different neurobiological, clinical and sociodemographic markers, but also offer perspectives for evidence-based, preventive early detection strategies. Preventive and precision medicine can be facilitated with the help of artificial intelligence, of the use of age-and time adequate tools such as social media and other awareness campaigns. Improving our understanding of what characterizes CHR individuals and of the factors that imply being “at-risk” for psychosis and/or for reduced psychosocial functioning—such as sexual di-/polymorphism, neuroplasticity, cognitive functioning, vegetative functions, clinical factors, etc.—may help to promote the development of novel interventions aiming to prevent transition into a chronic and difficult-to-treat disorder.

## Author Contributions

CM wrote the first draft of the Editorial. All other authors critically revised the draft and contributed to it. The final version was accepted by all authors.

## Conflict of Interest

The authors declare that the research was conducted in the absence of any commercial or financial relationships that could be construed as a potential conflict of interest.

## Publisher's Note

All claims expressed in this article are solely those of the authors and do not necessarily represent those of their affiliated organizations, or those of the publisher, the editors and the reviewers. Any product that may be evaluated in this article, or claim that may be made by its manufacturer, is not guaranteed or endorsed by the publisher.
